# Geometric Parameter Identification of Medical Robot Based on Improved Beetle Antennae Search Algorithm

**DOI:** 10.3390/bioengineering9020058

**Published:** 2022-01-29

**Authors:** Bin Kou, Dongcheng Ren, Shijie Guo

**Affiliations:** 1 Academy for Engineering and Technology, Fudan University, Shanghai 200433, China; 18110860041@fudan.edu.cn (B.K.); 18110860025@fudan.edu.cn (D.R.); 2 Guanghua Lingang Engineering Application and Technology R & D (Shanghai) Co., Ltd., Shanghai 201306, China

**Keywords:** beetle antennae search algorithm, random wandering, medical robot, localization accuracy

## Abstract

To improve the accuracy of common intelligent algorithms when identifying the parameters of geometric error in medical robots, this paper proposes an improved beetle antennae search algorithm (RWSAVSBAS). We first establish a model for the kinematic error in medical robots, and then add the random wandering behavior of the wolf colony algorithm to the search process of the beetle antennae search algorithm to strengthen its capability for local search. Following this, we improve the global convergence ability of the beetle antennae search algorithm by using the simulated annealing algorithm. We compare the accuracy of end positioning of the proposed algorithm with the frog-jumping algorithm and the beetle antennae search algorithm with variable step length through simulations. The results show that the proposed algorithm has a higher accuracy of convergence, and can significantly improve the accuracy of end positioning of the medical robot.

## 1. Introduction

Advances in Artificial Intelligence have penetrated into all aspects of healthcare in recent years, such as the combination of deep learning with smartphones for the remote monitoring of human activities to provide medical assistance in telemedicine, as reported by Wen et al., and the use of long-short term memory-Recurrent Neural Networks models in surgical robots to enhance their remote manipulation capabilities. Such approaches can improve the quality of treatment offered to patients [1,2].

Medical robots are a landmark product of Artificial Intelligence in medicine, and have the advantages of stability and flexibility. The use of medical robots can substantially improve accuracy and controllability during surgical operations [3]. It also eases the burden on medical personnel; in specific cases, medical robots can help ensure the safety of medical personnel, because they are resistant to radiation. They can also reduce the operating costs of hospitals and, thus, indirectly improve patient care. However, the medical robot itself has many emergency stop conditions, and its accuracy of motion is easily affected by changes in the environment due to the limitations of sensors [4,5]. With the increasing complexity of clinical surgical operations, the positioning accuracy of medical robots needs to be higher as it directly affects patient care and medical outcomes. Little research has been performed to address this issue. This study investigates the positioning accuracy of medical robots.

Positioning error in the operation of the robot mainly originates from errors in the assembly and manufacture of its parts. Software-based methods are the major means of reducing the positioning error of the robot [6,7]. Compensating for this error consists of four steps: modeling the kinematic error, measuring the error in data, identifying the geometric linkage parameter, and error compensation. Once the error model of the robot has been established, commonly used methods of parameter identification include the least-squares method. Wang et al. used the least-squares method to identify the accuracy of end positioning of medical robots by means of simulations [8,9]. In recent years, many researchers have identified geometric errors in robots by using intelligent algorithms, such as Chen et al., who used the particle swarm algorithm. Experimental results proved the effectiveness of their method [10]. Zhao et al. improved the accuracy of absolute positioning of the tandem robot by improving the genetic algorithm used for it [11]. However, the least-squares method is susceptible to noise, while the particle swarm algorithm tends to fall into the global optimum in later stages, and the coding of the genetic algorithm is complicated. The beetle antennae search (BAS) algorithm has been proposed in recent years, and has the advantages of fast iterations and high accuracy of convergence. For optimizing complex functions, however, the accuracy of convergence of BAS is low [12,13,14]. In this paper, we combine the wandering behavior of the wolf pack algorithm (WPA) with the simulated annealing algorithm and BAS to propose a randomized wandering simulated annealing-based variable-step-length beetle antennae search algorithm (RWSAVSBAS). We use it to identify geometric errors in medical robots to improve their accuracy of localization.

In subsequent sections, we first establish the operational science model of the BH-7 robot, then analyze the principle of RWSAVSBAS and its steps in geometric error calibration of medical robots, and finally, through simulation experiments, the RWSAVSBAS and the Variable-Step Beetle Antennae Search Algorithm (VSBAS) proposed in this paper and Shuffled Frog Leading Algorithm (SFLA), and the experimental results show that the proposed algorithm has higher accuracy in recognition results and can significantly improve the end positioning accuracy of medical robots [15,16,17,18,19].

## 2. DH Model Building

**Figure 1:**
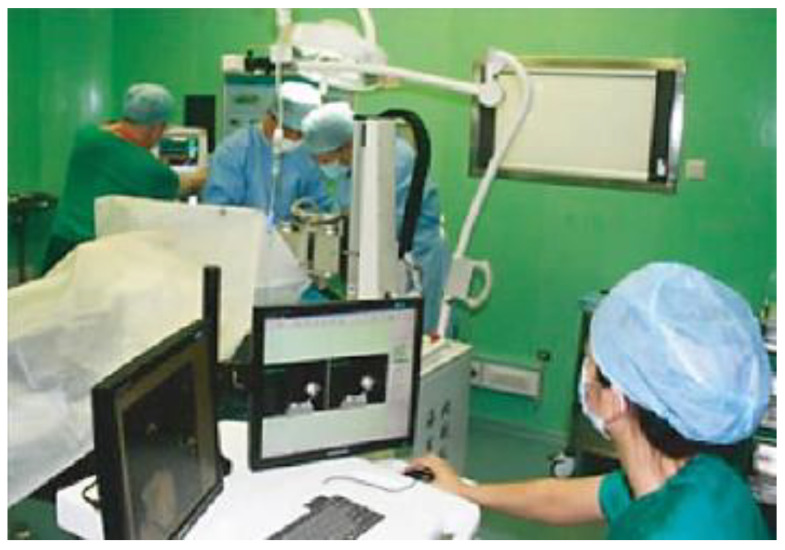
BH-7 robot.

We used the BH-7 medical robot for brain surgery as the experimental object [20,21,22]. Its core equipment consists of a positioning system that has been widely used in clinical practice, and offers visual recognition for remote surgical operations. The needle-piercing puncture at the end of the BH-7 robot can reach a specific location under the guidance of the vision system, and the robot works with the end-effector (needle-threading stab) that moves to the corresponding position under the action of a torque motor, as shown in Figure 1 below. The kinematics of this robot are modeled by the classical DH model. The robot has been used in a robotic system for positioning for brain surgery [23], and its accuracy was tested during the operation through the following steps:

(1) CT-scan the marker points and target points on the skull model.
(2) Initialization of BH-7 robot: Turn on the network cable and the power supply; open the visual recognition program, and use the stereo camera to calibrate the marker plates on each joint of the BH-7 robot in different states of motion to calculate error.
(3) Surgical planning: The CT images of the skull model from the hospital are reconstructed in 3D, and the appropriate number of marker points and target points are marked on the images.
(4) Marker point registration: The cranial model after the CT scan is placed in the working space of the BH-7 robot. The marker points are marked by the surgical planning program as well as the visual recognition program. The registration error of the marker points is then calculated.
(5) Stereotactic surgery by BH-7 robot: After marker point registration, simulated surgery is performed, and the mechanical arm of the BH-7 robot moves according to the simulated trajectory under the premise of trajectory safety. It points the puncture needle at the surgical target, at which time the distance between the real target and the manually measured puncture needle is the error in the accuracy of end positioning of the BH-7 robot.

**Figure 2:**
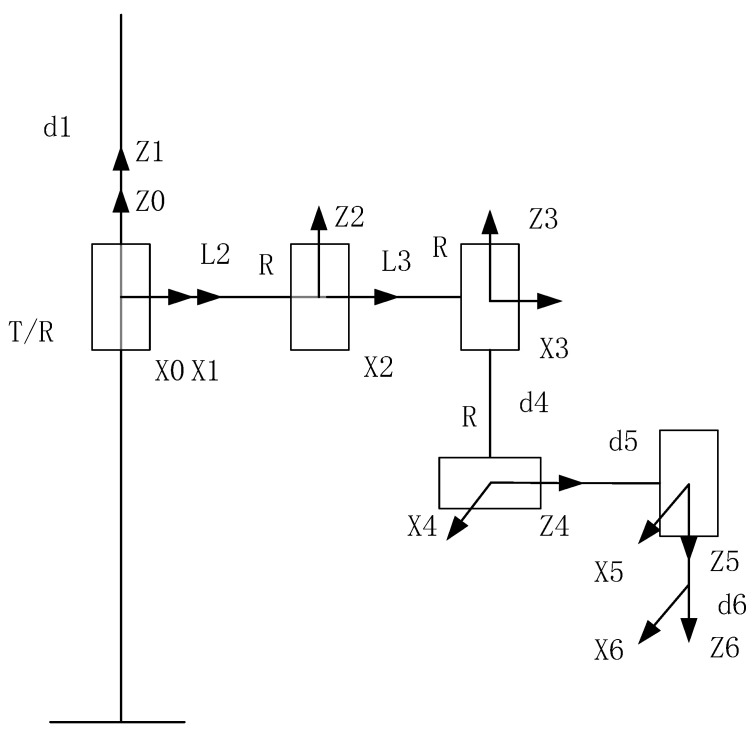
DH model of BH-7 robot.

Its kinematic model is shown in Figure 2, below.

As shown in Figure 2, the robot has five degrees of freedom. Except for the first joint, which is a moving joint, the remaining joints are rotating joints. The relevant DH parameters are shown in Table 1, below.

## 3. Improved Beetle Antennae Search Algorithm

Because the medical robot needs to identify more parameters of geometric error and the results of common VSBAS identification are poor [24], the improvements below are proposed for the BAS.

### 3.1. Improving Search Mode of BAS

The search mode of the BAS mainly relies on the left and right antennae. In the face of a more complex problem, such as calibrating the geometric parameters of medical robots, the accuracy of the BAS is often not sufficiently high because the left and right antennae have limited search space, and there is only one beetle in each search. The random wandering behavior of the WCA can strengthen the local search ability of the wolf pack algorithm, thus improving its accuracy of convergence. We add “random antennae”, inspired by the random wandering behavior of the WCA, to the left and right antennae of the BAS. In this improved BAS, each search process is equivalent to three antennae searching together. As the “wandering antennae” themselves are variable, the local search capability of the BAS is enhanced.

#### 3.1.1. Introduction to VSBAS

(1) The relationship between the antennae of the beetle is as follows:

(1) $$x_{l}-x_{r}=d_{0}.dir$$

In Equation (1), *x* represents the coordinates of the center of mass of the BAS, *x_l_* and *x_r_* represent its left and right antennae, and *d*_0_ represents the distance between them.

(2) The left and right antennae of the BAS can be expressed as follows according to their center of mass:

Left antenna:

(2) $$x_{l}=x+d_{0}.dir/2$$

Right antenna:

(3) $$x_{r}=x-d_{0}.dir/2$$

(3) For an *n*-dimensional spatial optimization problem, the direction of the antenna vector between the whiskers of the aspen is random, and can be expressed as:

(4) $$\vec{b}=\frac{rands(k,1)}{\left|rands(k,1)\right|}$$

In Equation (4) *k* denotes the number of dimensions of the variable and “*rands*” generates random numbers within the interval [−1, 1].

(4) The BAS determines the direction of movement of the antennae by comparing their fitness evaluations. The following equation is used:

(5) $$x=x-Step*dir*sign(f(x_{l})-f(x_{r}))$$

*Step* denotes the intended step, *sign*(.) is the sign function, and *f*(*x_l_*) and *f*(*x_r_*) are the adaptation values corresponding to the left and right antennae, respectively, so that if *f*(*x_l_*) > *f*(*x_r_*) during the iteration. The BAS then moves in the direction of its right antenna.

(5) The iterations of the BAS use a variable step rate, i.e.,

(6) Step=Step*eta

where *eta* denotes the adaptive factor of step size.

#### 3.1.2. WCA Random Wandering Behavior

The WCA is inspired by the behaviors of wolf packs: wandering, summoning, and siege. The head wolf has the best fitness value of the pack, where this is equivalent to the global optimal solution in the particle swarm. Wandering behavior refers to the behavior of a group of wolves with the best fitness value, other than the head wolf as scout wolves, searching randomly in the space.

(7) $$X_{id}^{p}=X_{id}+sin(2\pi \times P/h)\times step_{a}^{d}$$

In Equation (7), *h* denotes the total number of directions searched by a wolf, *P* denotes a direction (*p* = 1, 2, …, *h*), *X_id_* denotes the current position of the wolf in *d*-dimensional space, $X_{id}^{p}$ denotes its position after searching in direction *p* in the *d*-dimensional space, and $step_{a}^{d}$s the wandering step.

#### 3.1.3. Wandering Antennae Improvement

Inspired by Equation (7), the random wandering behavior of the WCA is added to the search pattern of the beetle antennae search algorithm, i.e., a “random antenna” is added to the original left and right antennae of the BAS. It is expressed as follows:

(8) $$Gbest1=Gbest+sin(2\pi \times P/h)\times d_{0}.dir$$

*Gbest* in Equation (8) denotes the global optimal solution of the BAS. That is, the BAS searches at each updated iteration, in addition to the original left and right antennae, while its global optimal solution is explored locally using random wandering behavior. As the value of the sign function changes with the number of iterations, the distance of detection of the random antenna is also constantly changing. In this way, in both the inner and the outer loops in the RWSAVSBAS search process, the search sizes of the left and right antennae of the BAS are reduced sequentially with the increase in the number of iterations according to Equation (6). However, from Equation (7), we see that the “random antenna” in the outer loop shows a periodic change in size according to the sign function, while the “random antenna” in the inner loop shows k periodic changes in size with changes in *Q*. These two ways of changing the step size enable the random antenna, and the left and right antennae to search together. This improves the local search ability of the BAS.

### 3.2. Improving Mechanism of Selection of Global Optimal Solution of BAS

However, as the BAS is a single particle search algorithm, it delivers poor results once it falls into the local optimum in the iteration process. The metropolis criterion of the SA can improve the ability of the BAS to emerge from the local optimal solution, and thus can improve its global search ability.

#### 3.2.1. Metropolis Guidelines

The simulated annealing algorithm determines whether to jump out of the local difference solution by virtue of the metropolis.

(1) Treat the randomly generated *x* as the optimal solution.
(2) Obtain a new solution near the initial solution *x_t_*, ∆*f* = *f* (*x_t_*) − *f* (*x*).
(3) Determine whether to choose the new solution *x_t_* by *min* {1, *exp* (−∆*f*/*T_k_*)} > random, where *T_k_* is the current temperature and *exp* is an exponential function with a natural number e as its base.

#### 3.2.2. Global Optimal Solution Selection Mode Improvement

The original BAS selects a new solution (*Gbest*1) each time by comparing it with the global optimal solution (*Gbest*) of the previous generation, and proceeds to the next iteration on the basis of merit. This causes the algorithm to prematurely develop. For RWSAVSBAS to obtain the maximum value, for example, when the global optimal solution *f* (*Gbest*) of the previous generation is smaller than the current solution *f* (*Gbest*1), the new solution is accepted.

If the previously generated global optimal solution, *f* (*Gbest*), is larger than the current solution, *f* (*Gbest*1), then the new solution is selectively accepted according to the metropolis criterion. This can help the BAS overcome the local optimum and improve its global search capability.

### 3.3. Calibration Process of Geometric Parameters

**Figure 3:**
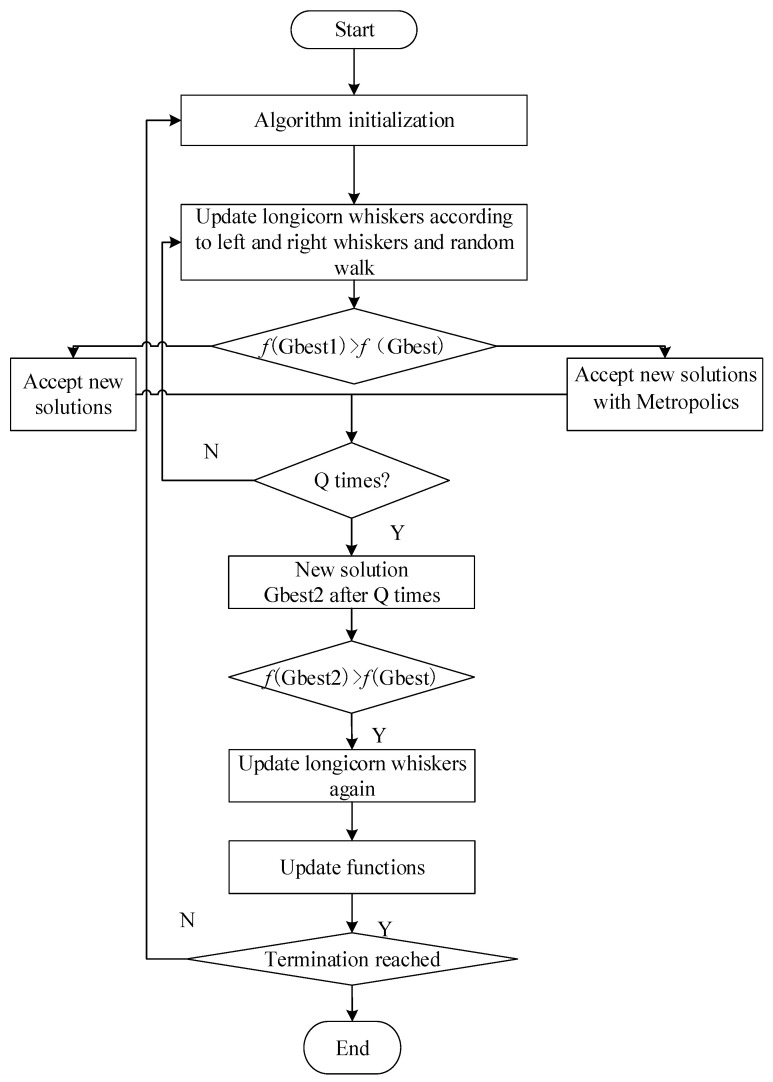
RWSAVSBAS algorithm flow.

The proposed algorithm is called the simulated annealing-based optimized variable-step-length beetle antennae search algorithm with randomized wandering (RWSAVSBAS), and its flow is as shown in Figure 3.

From Figure 3, it is clear that the steps to calibrate the geometric error when using the RWSAVSBAS for medical robots are as follows.

(1) Initialize the algorithm.
(2) Obtain the adaptation values of the left and right antennae of the algorithm, and of the “wandering antenna.”
(3) Update the step size of the algorithm according to Equation (5) to improve the accuracy of identification of the geometric parameters.
(4) The outer loop starts, and the algorithm enters the inner loop first, with *Q* cycles.
(5) The global optimal solution is selected according to the metropolis criterion of the simulated annealing algorithm.
(6) At the end of *Q* iterations of the inner loop, the search is performed again by probing the wandering antenna, and the left and right antennae. The global optimal solution is updated by merit.
(7) If the algorithm does not satisfy the termination condition, go to step 2; otherwise, the algorithm stops iterating and the corresponding parameters are used to calibrate the geometric error of the robot.

## 4. Experiments and Results

### 4.1. Posture Generation

The validity of the proposed algorithm is verified by the BH-7 robot, whose DH model parameters are shown in Table 1 [20]. The geometric parameter errors are shown in Table 2.

Twenty sets of theoretical joint angles are randomly and uniformly generated in the intervals of [−0.5,0.5] (unit: mm) and [−0.01,0.01] (unit: rad), and the previously set geometric errors are added to the nominal geometric parameters of the robot [25], and then the corresponding adaptation value formula is obtained by substituting the 20 sets of joint errors into the following Equation (8).

(9) $$f=\min(\sum_{i=1}^{N}\sqrt{(\;{\left(\delta P_{xi}\right)}^{2}+{\left(\delta P_{yi}\right)}^{2}+{\left(\delta P_{zi}\right)}^{2}})$$

*N* in Equation (9) denotes the number of medical robot error calibration points, *f* is the set of geometric parameter errors $\left(\Delta a_{i},\Delta d_{i},\Delta \alpha_{i},\Delta \theta_{i},\right)$ function, at different calibration points, to obtain the error between the nominal and actual positions of the medical robot, and then use RWSAVSBAS to solve $\left(\Delta a_{i},\Delta d_{i},\Delta \alpha_{i},\Delta \theta_{i},\right)$ the real value of the geometric error compensation is finally realized.

### 4.2. Results and Discussion

The experiments were conducted using MATLAB 9.1, setting the number of iterations *k* for each algorithm to 300, the step size *Step* for RWSAVSBAS to 0.05, the step size adaptive factor *eta* to 0.95, the initial annealing temperature ***T*** = 10,000 [26], the annealing factor *α* to 0.93, and the number of simulated annealing algorithm iterations *Q* to 100 for each iteration of BAS.

**Figure 4:**
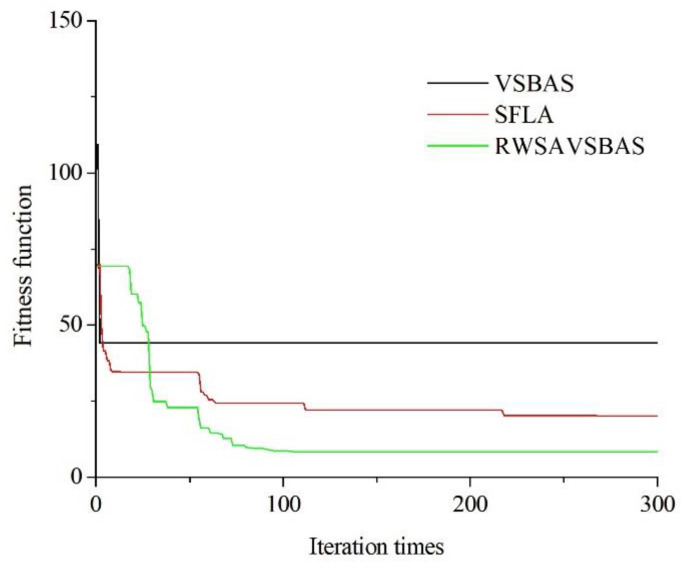
Evolution process figure.

Figure 4, below, shows the iteration profiles of each algorithm:

From Figure 4, it can be seen that RWSAVSBAS converges faster than SFLA and VSBAS in the pre-iterative stage and the final fitness function is smaller.

**Figure 5:**
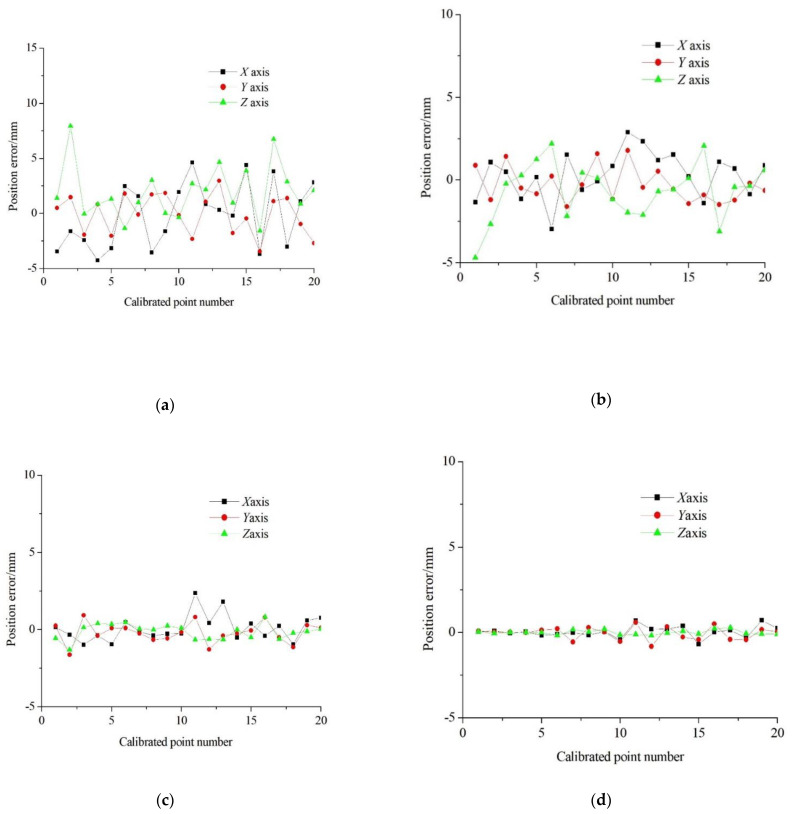
Comparison of error compensation results of each algorithm; (**a**) The error before compensation; (**b**) The error after compensation(VSBAS); (**c**) The error after compensation(SFLA); (**d**) The error after compensation(RWSAVSBAS).

Figure 5, below, shows the components of the end position error along the *X*, *Y*, *Z* axes before calibration of the medical robot. Figure 5 shows the *X*, *Y*, *Z* axis error components of the medical robot after calibration with VSBAS, SFLA and RWSAVSBAS.

The maximum absolute values of errors along the *X*, *Y* and *Z* axes before calibration are 4.61 mm, 2.96 mm and 7.93 mm respectively. The maximum absolute values of errors along the *X*, *Y* and *Z* axes are reduced to 2.97 mm, 1.77 mm and 3.13 mm after parameter compensation identified by VSBAS. c. The maximum absolute values of errors along the *X*, *Y* and *Z* axes are reduced to 2.36 mm, 1.64 mm and 1.32 mm after parameter compensation identified by SFLA. As can be seen from the graph in Figure 5, the absolute values of the maximum errors in the *X*, *Y* and *Z* axes are reduced to 0.71 mm, 0.82 mm and 0.26 mm after compensating for the parameters identified by SFLA. It can be seen that the RWSAVSBAS proposed in this paper has higher accuracy of calibration results compared with VSBAS and SFLA, which can significantly improve the end positioning accuracy of medical robots

## 5. Conclusions

(1) In this paper, a new improved Beetle Antennae Search Algorithm—RWSAVSBAS—is proposed, which combines the iterative law of Beetle Antennae Search Algorithm and introduces the random wandering behavior of the Wolf Colony Algorithm with the simulated annealing algorithm. The simulation results show that the proposed algorithm can effectively improve the localization accuracy of the medical robot, and has the advantage of high accuracy compared with other common algorithms.

(2) The geometric linkage parameter error of the medical robot is the main source of its positioning accuracy error, and by obtaining the actual linkage parameters of the medical robot, the end positioning accuracy can be effectively improved.

(3) The research in this paper is firstly verified by simulation experiments only, and actual experiments are needed to verify the effectiveness of the algorithm in this paper. In addition, the geometric linkage error of the medical robot is only considered in this paper. In the actual application, the end positioning accuracy of the medical robot is also affected by non-geometric error factors such as temperature, electromagnetic interference, and self-weight, which need to be further investigated.

(4) The simulation experiments proposed in this paper are mainly applied to the offline geometric error compensation experiments of medical robots. In the further research process, the online geometric error compensation of industrial robots can be used to conduct online compensation experiments for the end positioning errors of medical robots to further improve the real-time error compensation of medical robots.

## Figures and Tables

**Table 1:** Theoretical parameters of robot.

Joint *i*	$\Delta a_{i}/\mathrm{mm}$	$\Delta \alpha_{i}/\mathrm{rad}$	$\Delta d_{i}/\mathrm{mm}$	$\Delta \theta_{i}/\mathrm{rad}$
1	0	0	0	0
2	200	0	0	0
3	200	0	0	0
4	0	−90	−170	−90
5	0	−90	145	0
6	0	0	150	0

**Table 2:** Robot DH parameter error.

Joint *i*	$\Delta a_{i}/\mathrm{mm}$	$\Delta \alpha_{i}/\mathrm{rad}$	$\Delta d_{i}/\mathrm{mm}$	$\Delta \theta_{i}/\mathrm{rad}$
1	0.36	−0.0072	0.13	0.0093
2	0.45	0.0064	−0.45	−0.0081
3	−0.06	0.0083	0.40	0.0
4	0.24	0.0058	0.12	0.0076
5	0.13	−0.0097	−0.39	−0.009
6	−0.17	−0.0053	0.13	0.006

## Data Availability

The data used in this astudy were self-tested and self-collected during the test. As the control method in this paper is still being further optimized, the data cannot be shared at present. Therefore, data sharing is not applicable to this article.
